# Fourier transforms for fast and quantitative Laser Speckle Imaging

**DOI:** 10.1038/s41598-019-49570-7

**Published:** 2019-09-16

**Authors:** J. Buijs, J. van der Gucht, J. Sprakel

**Affiliations:** Physical Chemistry and Soft Matter, Wageningen University & Research, Stippeneng 4, 6708WE Wageningen, The Netherlands

**Keywords:** Fluid dynamics, Imaging techniques, Imaging techniques, Soft materials, Optical techniques

## Abstract

Laser speckle imaging is a powerful imaging technique that visualizes microscopic motion within turbid materials. At current two methods are widely used to analyze speckle data: one is fast but qualitative, the other quantitative but computationally expensive. We have developed a new processing algorithm based on the fast Fourier transform, which converts raw speckle patterns into maps of microscopic motion and is both fast and quantitative, providing a dynamnic spectrum of the material over a frequency range spanning several decades. In this article we show how to apply this algorithm and how to measure a diffusion coefficient with it. We show that this method is quantitative and several orders of magnitude faster than the existing quantitative method. Finally we harness the potential of this new approach by constructing a portable laser speckle imaging setup that performs quantitative data processing in real-time on a tablet.

## Introduction

Laser Speckle Imaging (LSI) is a versatile method to quantify and visualize the internal dynamics in complex and opaque materials. Initially devised as a medical imaging technique to map blood flow patterns beneath the skin^1–3^, LSI has emerged in recent years as a quantitative tool in material science^4–6^. Recent applications of the method include the quantification of flow patterns, the study of spatially-inhomogeneous Brownian motion and a non-invasive and complete micromechanical analysis in single micrometric pixels^7–11^. LSI operates on the basis of multiple light scattering, which occurs in highly opaque materials, and does not require labeling of the material of interest. Its suitability to probe processes in opaque materials offers substantial benefits over single-scattering or fluorescence based methods, which rely on optical transparency and therefore preclude the study of a wide range of practical materials. This makes LSI suitable for the study of dynamical processes in a wide variety of scenarios, ranging from solid-liquid transitions and crack patterns in drying films and droplets^10^, quantitative analysis of the mechanisms of fracture and self-repair^11,12^, to structural rearrangements in granular materials^13^.

LSI derives the internal dynamics in an opaque material by quantifying the intensity fluctuations of multiply scattered light. When a turbid material is illuminated by a coherent light source, the photons injected into the material undergo a large number of scattering events before exiting and traveling to the detector. Due to the numerous scattering events, the photon paths and polarization are completely randomized and can be described by diffusive statistics. Path length differences between photons that have traveled through the material lead to a characteristic interference, or speckle pattern. Motion of the scattering elements within the material changes the interference conditions, and thus leads to intensity variations that are analyzed to ascertain the rate and type of motion that has occurred. In Diffusing Wave Spectroscopy (DWS), a single speckle is detected to probe the average dynamics within the material^14^, while the entire speckle pattern is collected in LSI to gain spatially-resolved information.

To go from the raw data to a measure for the dynamics, various approaches are available. In medical imaging, a measure for the spatial or temporal variance of a set of speckles is most commonly used to calculate the speckle contrast; this method is often denoted as Laser Speckle Contrast Analysis (LASCA)^15–17^. While this approach can not easily be applied to quantify different dynamical processes at different frequencies can not easily be applied to quantify different dynamical processes at different frequencies, it is used because the computational efficiency is high. This has enabled real-time data treatment, which is crucial in a clinical setting^18,19^. Efforts to obtain quantitative data from LASCA have been successful but at the cost of slowing the method down to the point where real-time data analysis is not feasible^20^. Quantitative LASCA requires sets of speckle images with a range of exposure times, thereby increasing data acquisition time by at least a factor of ten, and introduces an additional curve-fitting step.

Quantitative LSI, such as often used in material science scenarios^7–11^, requires the measurement of dynamics over a wide frequency range, so that different dynamical processes (such as Brownian motion, flow, and deformation) can be distinguished and quantified. This is usually requires the measurement of dynamics over a wide frequency range, so that different dynamical processes (such as Brownian motion, flow, and deformation) can be distinguished and quantified. This is usually performed by calculating the autocorrelation function of the temporal intensity trace^21^. This approach is identical to data treatment in DWS, thus benefiting from established DWS theory to obtain quantitative data, such as the mean-squared displacement of scattering particles, from which diffusion coefficients and flow rates can be extracted^22–26^, or to obtain a spatially-resolved invariant of the strain tensor to enable strain field visualisation^12,27,28^. While quantitative, this approach is computationally intensive and not suited for real-time LSI. The current trade-off between computational efficiency and the level of detail in the information gained for an LSI experiment is harsh. This poses the need for a universal analysis method that is both quantitative over a wide frequency range over a wide frequency range and fast.

In this paper we propose a new method for LSI analysis that uses Fourier transformations (FT) to acquire the power spectral density to quantify speckle fluctuations in the frequency domain^29^. According to the Wiener-Kinchin theorem the power spectral density and autocorrelation function contain the same quantitative information^30,31^. However, the power spectrum can be calculated in a fraction of the time, owing to the very efficient Fast Fourier Transform (FFT) algorithm^32^. Therefore the same quantitative information can be obtained much faster. Once the power spectrum has been calculated, the autocorrelation function can be readily obtained as its Fourier transform. Once the power spectrum has been calculated, the autocorrelation function can be readily obtained as its Fourier transform. The power spectral density of speckle intensity fluctuations has been calculated before, however not with the aim to spatially visualize dynamics^33,34^. For DWS the FFT has been used to calculate the correlation function efficiently, but no information was extracted from the power spectrum directly^35,36^. Therefore a framework to extract quantitative results from the power spectral density does not exist.

In the following, we will present a framework for the extraction of quantitative data from Fourier transform LSI (FT-LSI) experiments. We benchmark the performance of this approach to both LASCA and conventional LSI, and confirm its validity by measuring the diffusion coefficients of Brownian particles. We find that for big data sets FT-LSI, is several orders of magnitude faster than LSI. The result of the FT-LSI is a full power spectrum for every pixel which holds significantly more information than the single speckle contrast value obtained with LASCA. The combination of computational efficiency and access to the entire the power spectrum, enables result display in the form of a spectrogram, which gives a full overview of the power spectral density as function of both time and frequency. We calculate a spectrogram for the scenario of a short flow pulse through a micro-channel and show how it enables direct observation of both flow start-up and cessation within a microchannel. Finally, we show that the strongest advantage of this method is its ability to perform real-time quantitative analysis. To demonstrate this, we build a portable LSI set-up which is controlled by a tablet and develop software that performs the FT-LSI in real-time. We use this set-up to study the heat welding of plastic layers during 3D printing.

## Results

### Theory

Traditionally, quantitative LSI starts from the intensity autocorrelation function *g*_2_, as a function of the lag time $\tau$. Speckle images, separated in time by $\tau$, are correlated to obtain a map of local dynamics. The *g*_2_ autocorrelation function is computed as

1 $${g}_{2}(t,x,y,\tau )=\frac{\langle I(t,x,y)\cdot I(t+\tau ,x,y)\rangle }{\langle I(t,x,y)\rangle \,\langle I(t+\tau ,x,y)\rangle }$$

To increase statistics, averaging can be performed in time and/or space, but at the cost of spatial and/or temporal resolution. Using the Siegert relation the *g*_2_ can be converted to field autocorrelation function *g*_1_

2 $${g}_{1}(\tau )=\frac{1}{\sqrt{\beta }}\cdot \sqrt{{g}_{2}(\tau )-1)}$$

where *β* is a instrumental constant, which is determined in experiments from the extrapolated intercept of *g*_2_ to $\tau =0$. A common route to obtain quantitative information from these correlation functions, is to extract the strain and the mean squared displacement of the scatterers^12,13,37^:

3 $${g}_{1}(\tau )=\exp [-\,2\gamma {k}_{0}{l}^{\ast }\sqrt{3f(U)+\langle \Delta {r}^{2}(\tau )\rangle /{l}^{{\ast }^{2}}}]$$

where *γ* is a numerical constant, ${k}_{0}=2\pi n/\lambda$ is the wavevector, with refractive index *n* and wavelength *λ*, l* is the photon transport mean free path, *f*(*U*) an invariant of the strain tensor and $\langle \Delta {r}^{2}(\tau )\rangle$ the mean squared displacement. For the simple case of scatterers that perform purely diffusive motion in a material free of mechanical deformations, the diffusion coefficient *D* can be obtained from $\langle \Delta {r}^{2}(\tau )\rangle =6D\tau$, and the equation above simplifies to:

4 $${g}_{1}(\tau )=\exp -\sqrt{\kappa |\tau |}$$

where $\kappa ={\gamma }^{2}{k}_{0}^{2}6D$. This equation can be generalized to non-diffusive dynamics, such as directional flow or sub-diffusive motion in densely packed materials. In conventional LSI, this can be done by introducing a stretching parameter *α* in the exponent

5 $${g}_{1}(\tau )=\exp \,[\,-\,|\kappa \cdot \tau {|}^{\alpha }]$$

where *α* describes the nature of the motion: *α* is $\tfrac{1}{2}$ for diffusive motion, 1 for ballistic motion and <$\tfrac{1}{2}$ for sub-diffusive processes. Note that Eq. 4 is equal to Eq. 5 with $\alpha =\tfrac{1}{2}$.

The Wiener-Kinchin theorem^30,31^ states that the same information is accessible in the frequency domain. Such an approach benefits from very efficient fast Fourier transform (FFT) algorithms. With slight modifications to the theory, the same quantitative data can be extracted. We start with the analytical Fourier Transform of Eq. 5 which according to the Wiener-Kinchin theorem is equal to the power spectrum. For low frequencies this gives:

6 $$\mathop{\mathrm{lim}}\limits_{\omega \to 0}\,P(\omega )=\frac{2}{\kappa }\cdot \Gamma (1+\frac{1}{\alpha })$$

and for high frequencies:

7 $$\mathop{\mathrm{lim}}\limits_{\omega \to \infty }\,P(\omega )\approx \frac{2{\kappa }^{\alpha }}{|\omega {|}^{1+\alpha }}\cdot \Gamma (1+\alpha )\cdot sin(\frac{\alpha \pi }{2})$$

These two limits are separated by a critical corner frequency ω*, that is found at:

8 $${\omega }^{\ast }={[\frac{\Gamma (1+\frac{1}{\alpha })}{\Gamma (1+\alpha )sin(\frac{\alpha \pi }{2})}]}^{-\frac{1}{1+\alpha }}\cdot \kappa $$

For the simplest case of purely diffusive motion of the scatterers $\alpha =\tfrac{1}{2}$, the analytical form of the power spectrum reads:

9 $$\begin{array}{rcl}P(\omega ) & = & \sqrt{\frac{\pi \kappa }{2}}\cdot \frac{1}{|\omega {|}^{\frac{3}{2}}}\{cos(\frac{\kappa }{4|\omega |})[1-2C(\sqrt{\frac{\kappa }{2\pi |\omega |}})]\\  &  & +\,sin(\frac{\kappa }{4|\omega |})\,[1-2S(\sqrt{\frac{\kappa }{2\pi |\omega |}})]\}\end{array}$$

which uses two Fresnel integrals

10 $$C(x)={\int }_{0}^{x}\,cos({t}^{2})dt\,{\rm{and}}\,S(x)={\int }_{0}^{x}\,sin({t}^{2})dt$$

At low frequencies the power spectrum converges to a horizontal asymptote described by

11 $$\mathop{\mathrm{lim}}\limits_{\omega \to 0}\,P(\omega )=\frac{4}{\kappa }$$

while at high frequencies the power spectrum converges to

12 $$\mathop{\mathrm{lim}}\limits_{\omega \to \infty }\,P(\omega )\approx \sqrt{\frac{\pi \kappa }{2}}\cdot \frac{1}{|\omega {|}^{\frac{3}{2}}}$$

These two limits are separated by a critical corner frequency ω*

13 $${\omega }^{\ast }=\frac{1}{2}\cdot {(\frac{\pi }{4})}^{\frac{1}{3}}\cdot \kappa $$

**Figure 1:**
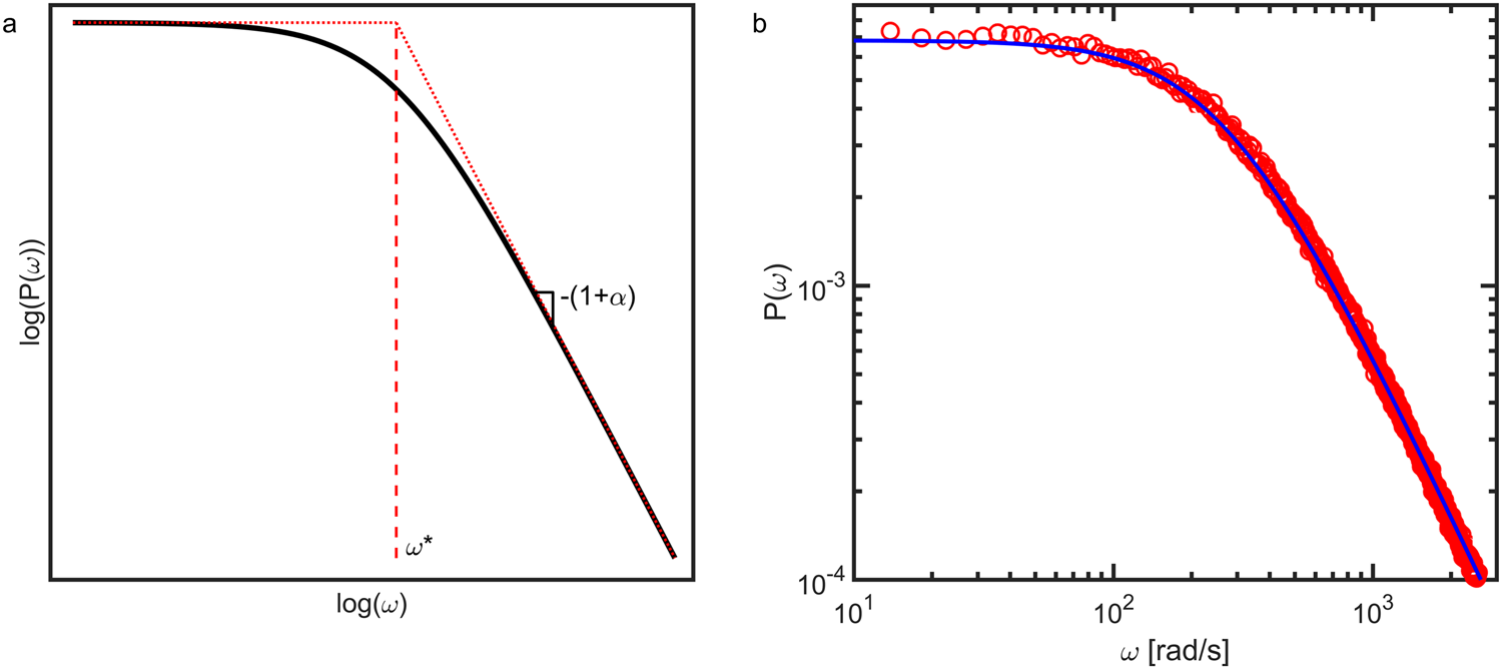
(**a**) Theoretical power spectrum of speckle intensity fluctuations, described by Eqs 6–8. The critical frequency ω* is obtained from the intercept between the fit of two straight lines through the low and high frequency domains. The type of dynamics, described by *α*, can be obtained from the slope of the high frequency domain. (**b**) Experimental power spectrum () of the diffusion of 1 *μ*m polystyrene spheres in a 50/50 mixture of water and glycerol. The power spectrum is also computed by taking the Fourier transform of the autocorrelation function of the same raw speckle data ().

On a log-log scale, both the high and low frequency domains approach a straight line, as is shown in Fig. 1a. Equations 11–13 can be used to determine the diffusion coefficient from an experimental power spectrum. From the slope of the high frequency domain we can determine *α*, which gives insight into the nature of the scatterer motion. Since the slope is equal to −(1 + *α*), we have a slope of −$\tfrac{3}{2}$ for diffusive motion and a slope of −2 for ballistic motion, in agreement with previous results^38,39^. For diffusive motion, the diffusion coefficient can be extracted both from the frequency-independent plateau at low frequencies (11 and 6) and from the corner frequency (13 or 8).

It should be noted that this analysis is valid only for ergodic samples. As shown previously^40^, the presence of a non-fluctuating component in the scattered light modifies the correlation function *g*_2_ and complicates the analysis. Since the power spectrum is directly related to *g*_2_, it is equally affected by non-fluctuating scatterers. An approach to correct for non-ergodicity was presented by by Zakharov *et al*.^41^, who directly measured the fraction of non-mobile scatterers in the sample. A similar work-around can be applied for FT-LSI. However, in this paper we will only consider ergodic samples, for which such corrections are not needed.

### Validation

To verify the predictions made above, we study a sample of diffusing colloidal particles in a simple viscous solvent. We take 1 *μm* polystyrene spheres suspended in a 50/50 *wt*% mixture of glycerol and water. The cuvette is placed in a backscatter LSI instrument, which is described in the methods section which is described in the methods section, and $N={10}^{5}$ images are collected at a rate of 10^4^ images per second.

The power spectrum of the speckle intensity fluctuations is calculated using two approaches. First, the FFT algorithm is used to transform the intensity fluctuations per speckle to the frequency domain. The resulting amplitudes are squared to obtain the power spectrum and averaged over all speckles in the field of view. Further details regarding the procedure and normalization are provided in the methods section. Secondly, the *g*_1_ autocorrelation function is calculated in real space, according to Eqs 1 and 2, interpolated with an exponential decay which is then Fourier transformed to the frequency domain.

The power spectra obtained in these two approaches are identical within experimental noise, as shown in Fig. 1b. This confirms the validity of the Wiener-Kinchin theorem for this method. Moreover, the experimental power spectra (Fig. 1b) have the same shape as that predicted by our theoretical analysis (Fig. 1a). This is the first confirmation that Fourier analysis can be applied to obtain quantitative data from LSI experiments.

As further validation, we perform a set of experiments on colloids diffusing in solvents of varying viscosity. We prepare suspensions of 1 *μm* polystyrene spheres in glycerol:water mixtures containing between 0 to 60 wt% glycerol. The diffusion coefficient can be computed using the Stokes-Einstein relation:

14 $$D=\frac{{k}_{B}T}{6\pi \eta r}$$

with *k*_*B*_*T* the thermal energy, $\eta$ the fluid viscosity and *r* the particle radius.

**Figure 2:**
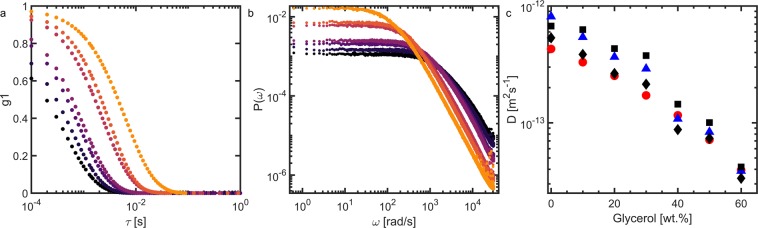
(**a**) Correlation functions calculated for the diffusion of 1 *μm* polystyrene particles in a water-glycerol mixture. The dark color corresponds to 0% glycerol, which increases with steps of 10% to a maximum of 60% for the lightest series. (**b**) Power spectral densities calculated for the diffusion of 1 *μm* polystyrene particles in a water-glycerol mixture. The dark color corresponds to 0% glycerol, which increases with steps of 10% to a maximum of 60% for the lightest series. (**c**) Diffusion coefficients calculated with Stokes-Einstein (), LSI () and FT-LSI using the critical frequency (◾) and the height of the plateau (♦).

Also here, we collect $N={10}^{5}$ speckle images at a rate of 10^4^ images per second for every sample. The corresponding *g*_1_ curves are shown in Fig. 2a and power spectra obtained by direct FFT in Fig. 2b. We note that all power spectra exhibit the predicted *ω*^−3/2^ slope in the high frequency limit. Both the correlation functions and power spectra exhibit the expected trends. The decorrelation is shifted to larger correlation times as the glycerol concentration increases (Fig. 2a) and the power spectra show an increase in the low-frequency plateau value and a decrease in the corner frequency ω*, as predicted above.

Diffusion coefficients are obtained from the real-space correlation curves using Eq. 3. For the FT-LSI data, we determine the corner frequency ω* as the intersection between two fits through the low frequency plateau and high-frequency power law regimes. In this FT-LSI approach, we determine diffusion coefficients both from the height of the plateau, using Eq. 6, as well as from ω* on the basis of Eq. 8. We find a good agreement between the calculated diffusion coefficients and those obtained in the conventional approach and the two methods emerging from the FT-LSI analysis. This lends further confirmation that FT-LSI can yield accurate and quantitative measurements.

### Computational efficiency

The main advantage of the FT-LSI approach is its computational efficiency. To benchmark this, we compare the time it takes to compute a spatially-resolved LSI map using three different methods: i) LASCA, which is fast but not quantitative, ii) LSI, which is quantitative but slow and iii) FT-LSI which we will show is as fast as LASCA, and as shown above is as quantitative as conventional LSI.

LASCA estimates a measure for the rate of scatterer motion from the blurring of speckles by computing their intensity variance. It uses the contrast function *K*, defined as:

15 $$K=\frac{\sigma }{\langle I\rangle }$$

where *σ* is the standard deviation of the intensity of a speckle over time and $\langle I\rangle$ is its temporal average.

In quantitative LSI, the raw data is converted into an intensity-intensity autocorrelation function (Eq. 1) as a function of lag time $\tau$, which is limited at the low end by the acquisition rate and at the high end by the length of the time series. To increase the statistical reliability of the data, averaging in space and/or time is often performed, at the cost of spatial and/or temporal resolution.

FT-LSI uses a direct conversion of the raw speckle data into the frequency domain using fast Fourier transform routines (as described in detail in the methods section). The result is squared and normalized to obtain the power spectrum.

**Figure 3:**
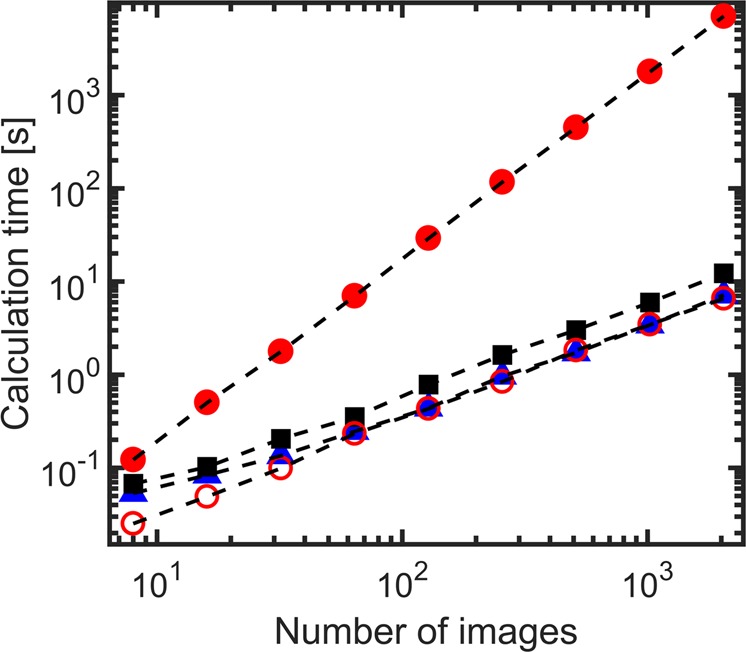
Calculation time as a function of data-set size with the amount of images containing 640 × 480 pixels each. Each calculation was repeated ten times and averaged, however variations were minimal. The three methods we compare are LSI (full correlation , single $\tau$ correlation ), LASCA () and FT-LSI (full spectrum ◾).

We compare the computational efficiency of these three approaches (Fig. 3). We see how the conventional approaches differ widely in their efficiency; LASCA, performed on a single correlation time equal to the inverse of the frame rate, is substantially faster than the computation of the full autocorrelation function, but contains only qualitative data. By contrast, our FT-LSI approach results in data with the same information density as LSI, but operates as fast as LASCA. The calculation time t of FT-LSI and LASCA scales as $t\propto N\,\log \,N$, with the number of images *N*, compared to $t\propto {N}^{2}$ for LSI. It should be noted that multi-tau correlation schemes have been developed for DWS, which speed up calculation of the correlation function significantly. In such schemes, the correlation function is calculated not for all correlation times, but for a sequence of logarithmically spaced correlation times, with reduced time averaging to obtain correlation curves with negligible loss of accuracy^42^. Such correlation schemes have not yet been applied to LSI, but would obviously speed up correlation LSI. A lower limit for the calculation time for correlation LSI is the time needed to compute the correlation for a single $\tau$. This is slightly faster than FT-LSI and comparable to LASCA (Fig. 3), but only yields information for a single correlation time for each pixel. Obviously, the computation time of multi-tau schemes will increase as a wider frequency range must be probed, and for these cases FT-LSI is a very attractive alternative. The computational efficiency of FT-LSI thus brings real-time quantitative imaging within reach, even for very large data sets.

### Flow imaging

We have shown above how FT-LSI can be used to quantify simple Brownian motion. To explore the range of scenarios in which FT-LSI can be applied, we now consider the case of directional flow in a Brownian suspension. Traditional LSI and LASCA have been used extensively to study blood flow patterns in a medical context^1,2,17,43–49^. But the use of LSI to image flow fields could also be of interest in other scenarios, such as the real-time monitoring of industrial production processes that involve flow of complex fluids.

Convective processes in fluids that contain Brownian particles contain a mixture of different types of motion, which is often spatially and temporally inhomogeneous. To reduce this complexity, a spatially-averaged spectrogram, generated by FT-LSI is a convenient starting point as it gives a complete overview of the temporal evolution of the entire frequency-dependent response of the fluid of interest, for example to identify transitions between motion dominated by convective and diffusive motion.

**Figure 4:**
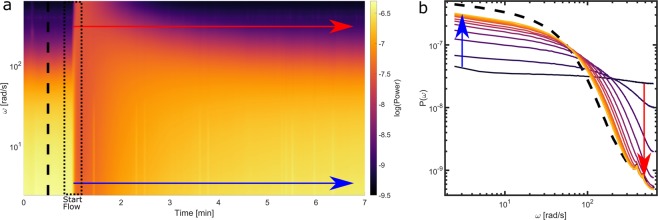
(**a**) A spectrogram of flow in a channel to explore the time-frequency domain of the measurement. There is no flow in the first minute and the system is in equilibrium. At t = 1 min we apply a 6 second flow burst after which the channel slowly goes back to equilibrium. (**b**) Evolution of the power spectrum as the flow channel goes back to equilibrium. The dotted line is the power spectrum at t = 30 seconds (equilibrium). After the start of the pressure pulse, power spectra are shown for 30 second intervals. In both figures the blue arrow shows the power evolution of the low frequencies and the red arrow the power evolution of the high frequencies.

To explore this, we create a simple microchannel that is created from a sacrificial template in PDMS (details described in the methods section) and loaded with a dispersion of titanium dioxide particles in a 90/10 *wt*% glycerol-water mixture. The suspension is left to equilibrate in the channel in the absence of flow, and we begin our FT-LSI recording. One minute after the imaging starts, we apply a pressure pulse, corresponding to an imposed flux of 100 *μ*L/min that lasts for 6 seconds. After this, the pressure is switched off and we continue recording how the convective motion relaxes back to a quiescent state in which Brownian motion dominates. We compute the full spectrogram for this experiment, which is shown in Fig. 4a, and extract representative power spectra at 30 second intervals, as shown Fig. 4b.

The spectrogram shows a sharp transition at the time of the pressure pulse, where the motion of the scatterers transitions from diffusive motion to directional flow. In agreement with the theoretical predictions above, this change in motion causes the power of low frequencies to decrease and that of high frequencies to increase. When the pressure is removed, the relaxation of the convective flow to the quiescent state is much more gradual, and takes several minutes to complete.

Extracting power spectra at selected time points enables a more detailed view on the process (Fig. 4b). We observe that the transition from convective to Brownian motion in the relaxation regime of the experiment follows the trends predicted by the theoretical framework, with a plateau that shifts upwards as convective motion ceases, while the slope of the high-frequency limit goes from ≈−2 to −3/2. Within this approach, quantitative visualization of even complex flow scenarios can be performed with relative ease.

### Portable real-time FT-LSI

The FT-LSI method paves the way to quantitative real-time imaging as the procedure can resolve spectrally-decomposed maps of scatterer dynamics at high rates. This could be particularly advantageous in clinical settings, where a fast diagnosis is beneficial. Such real-time LSI also benefits time-sensitive experiments as it allows rapid adjustments to the measurements *in*-*situ*. Furthermore it results in substantial data-reduction, as it does not require storage of all raw speckle images. Several real-time approaches based on the qualitative LASCA routine have been proposed^18,19,33^, however real-time quantitative LSI was not possible to date.

To resolve this, we have developed software that performs quantitative FT-LSI in real-time with minimal computational requirements, such that it can be performed on a simple tablet computer, which is connected to a portable LSI set-up. This is inspired by recent advances in portable LSI setups^43,50^, that enable measurements outside the laboratory.

**Figure 5:**
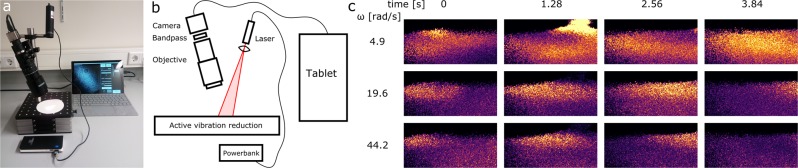
(**a**) Photograph of the portable LSI setup. (**b**) Schematic drawing of the portable LSI setup. (**c**) FT-LSI images collected with the portable LSI setup of molten ABS plastic extruded from a 3D printer. The extruder moves from left to right and deposits a very thin layer on the already printed material. Light colors correspond to high power, dark colors to low power.

A photograph of the portable and cordless LSI setup is shown in Fig. 5a and a schematic drawing in Fig. 5b. The sample is illuminated with a 50 mW laser diode (*λ* = 685 nm, CNI lasers) which is powered from a power bank. The beam is expanded by a single lens in front of the laser. Speckle patterns are collected with a simple CMOS camera (Thorlabs), equipped with an objective with variable zoom and iris (Navitar). The ambient light is removed with a notch filter, whose transmission band is centered at the laser wavelength, placed between the camera and the objective. The setup is mounted on a compact platform with active vibration reduction (Accurion). The tablet controls the camera via USB and collects and processes the speckle images in a home-built application, coded in the python language. The software collects 50 speckle images per second with a resolution of 640 × 512 pixels. The power spectrum is continuously calculated for each pixel and visualized for one single frequency as a spatially-resolved power map. For data sets of 32 speckle images per power spectrum, we can calculate 4 FT-LSI images per second, which makes the experiment responsive to real-time adjustments.

As a demonstration, we use the portable FT-LSI setup to study the layer bonding process during 3D-printing. Molten ABS plastic is extruded from a heated nozzle and a thin layer is deposited on previously printed layers. To weld newly printed layers onto previously deposited material, which has cooled down on contact with the printing plate, heat transfer from the new layer into the existing structure is paramount. Entanglement of the polymers during this layer bonding process is critical for printing strong objects. We use FT-LSI to follow the 3D-printing process in real time and present the results in Fig. 5c. In these images, the nozzle moves from left to right and deposits a 200 *μ*m thick layer on a 3 mm high existing object. There is a clear increase in dynamics around the deposition area, which reflects an increase in the polymer fluidity as a result of the heat transport from the new layer into the existing structure. This effect is visible several seconds after the nozzle has passed and spreads to a depth of at least 3 mm, which is equal to 15 layers in this case. With FT-LSI the layer bonding process can be studied and followed in real time.

This example shows the ability of the FT-LSI set-up to perform in versatile situations. No modifications to the 3D-printer were needed and the portability of the FT-LSI set-up enabled it to be placed inside the printer without issue.

## Discussion

In this paper we have presented a new FT-LSI analysis, which uses fast fourier transforms to obtain quantitative data with very high computational efficiency, offering orders-of-magnitude acceleration of quantitative data analysis. This advancement could be very benificial in the medical field, where current real-time approaches only give access to qualitative data. There also are a plethora of non-medical applications where this approach could become useful. For example, in understanding layer bonding in 3D printing, as exemplified above, in the monitoring of complex fluid flow patterns or in the micromechanical imaging of extreme mechanics^10–12^. LSI has become more versatile by combining the real-time analysis with a portable setup. Setup portability allows measurements of complex systems *in*-*situ* as well as monitoring adjustments directly. The miniaturization of imaging technologies like LSI is an ongoing process and there are many more challenges left to tackle. Tablets and phones already come with built in cameras; if these cameras can be used directly, with small built in LED-lasers, this could enable direct blood flow imaging at the consumer level.

## Methods

### Backscatter LSI

Laser Speckle Imaging measurements in the backscatter detection geometry are performed on a set-up that has been described in detail elsewhere^10,11^. For illumination a 532 nm laser (Cobolt Samba, 1W) is used. Two cameras were used on this set-up. To obtain 10^4^ images per second a high speed camera (Fastec, HiSpec 1) was used with a small field of view ($128\ast 8$). For the flow channel experiment a camera with live-streaming capabilities (Stemmer, Dalsa Genie) was used to stream images at 200 fps, with a resolution of $640\ast 480\,{\rm{pixels}}$ for a longer period. This instrument is used for all experiments, except when specified otherwise.

### FT-LSI routine

The FT analysis of LSI data starts by computing the power spectral density. We perform the FFT on a time sequence of *N* speckle patterns, where *N* is a power of 2 for computational efficiency. Each (*xyt*)-voxel is treated as an intensity time series of length *N*; all voxels are transformed simultaneously as a three dimensional matrix. First, we subtract the mean intensity for each voxel, to represent the time trace as intensity fluctuations around a mean of zero. This matrix is transformed to the frequency domain with the standard fft routine. Amplitudes corresponding to negative frequencies are discarded to obtain the single-sided amplitude spectrum, which is squared to obtain the power. The power is normalized by dividing by the frame rate and the trace lenght *N*, as well as dividing by $\langle {I}^{2}\rangle -{\langle I\rangle }^{2}$. The result is a three dimensional matrix $P(x,y,\omega )$ containing power spectral densities for each *xy* pixel. The frequencies *ω* are equally spaced between 0 and (π* frame rate), with *N*/2 + 1 unique frequencies. This matrix is either averaged spatially, or the result of a single frequency is mapped to a surface to obtain a power image for a particular frequency.

### Channel fabrication

The flow channel is created using the ESCARGOT method^51^. First the channel is 3d-printed (Ultimaker) with ABS plastic and the object is embedded in a silicone elastomer (Sylgard 184, Dow Corning), which is cured at 60 °C. Subsequently, the entire device is submerged in acetone to dissolve the ABS and leave a hollow channel in the silicone device. The channel has a rectangular cross-section with a width of 2.0 mm and a height of 600 *μm*. For the flow experiment, we introduce a suspension of of 2 wt% titania nanoparticles, in a 10/90 wt% water/glycerol mixture, using a syringe.

### Real-time FT-LSI

To enable real-time FT-LSI imaging, we have developed a software application in the Python 2.7 programming language. The program runs over three separate threads to allow parallel real-time data collection and processing.

The first (main) thread contains a graphical user interface (GUI) to control the acquisition and analysis settings. This GUI continuously displays the most recent FT-LSI image at a selected frequency, using the pygame module. The second thread continuously collects the most recent speckle image from the camera and places it in a circular buffer in the main thread. Optionally the raw speckle images are saved to disk for later analysis. The third thread continuously performs the FT-LSI algorithm. The analysis starts with the *n* most recent speckle images from the main thread and performs the FT-LSI routine described above. The power image, at a single frequency, is rescaled and displayed in a color heat map. Optionally, also analyzed images are also saved to disk, as well as the quantitative $P(x,y,\omega )$ data for later analysis.

## Data Availability

The developed software and data collected during the current study are available from the corresponding author upon reasonable request.

## References

[1] Fercher A, Briers JD (1981). Flow visualization by means of single-exposure speckle photography. Optics communications.

[2] Briers, J. D. Laser speckle contrast imaging for measuring blood flow. *Optica Applicata* **37** (2007).

[3] Briers JD, Richards GJ, He X-W (1999). Capillary blood flow monitoring using laser speckle contrast analysis (lasca). Journal of biomedical optics.

[4] Wintzenrieth F, Cohen-Addad S, Le Merrer M, Höhler R (2014). Laser-speckle-visibility acoustic spectroscopy in soft turbid media. Physical Review E.

[5] Nader CA (2016). Evaluation of low viscosity variations in fluids using temporal and spatial analysis of the speckle pattern. Optics letters.

[6] Hajjarian Z (2016). Laser speckle rheology for evaluating the viscoelastic properties of hydrogel scaffolds. Scientific reports.

[7] Zakharov P, Scheffold F (2010). Monitoring spatially heterogeneous dynamics in a drying colloidal thin film. Soft Materials.

[8] Amon A (2012). Hot spots in an athermal system. Physical review letters.

[9] Ansari MZ, Nirala AK (2016). Following the drying process of fevicol (adhesive) by dynamic speckle measurement. Journal of Optics.

[10] Van Der Kooij HM, Fokkink R, Van Der Gucht J, Sprakel J (2016). Quantitative imaging of heterogeneous dynamics in drying and aging paints. Scientific reports.

[11] van der Kooij HM, Susa A, Garca SJ, van der Zwaag S, Sprakel J (2017). Imaging the molecular motions of autonomous repair in a self-healing polymer. Advanced Materials.

[12] van der Kooij HM (2018). Laser speckle strain imaging reveals the origin of delayed fracture in a soft solid. Science advances.

[13] Amon A, Mikhailovskaya A, Crassous J (2017). Spatially resolved measurements of micro-deformations in granular materials using diffusing wave spectroscopy. Review of Scientific Instruments.

[14] Pine D, Weitz D, Zhu J, Herbolzheimer E (1990). Diffusing-wave spectroscopy: dynamic light scattering in the multiple scattering limit. Journal de Physique.

[15] Cheng H (2003). Modified laser speckle imaging method with improved spatial resolution. Journal of biomedical optics.

[16] Briers JD, Webster S (1996). Laser speckle contrast analysis (lasca): a nonscanning, full-field technique for monitoring capillary blood flow. Journal of biomedical optics.

[17] Draijer M, Hondebrink E, van Leeuwen T, Steenbergen W (2009). Review of laser speckle contrast techniques for visualizing tissue perfusion. Lasers in medical science.

[18] Yang O, Cuccia DJ, Choi B (2011). Real-time blood flow visualization using the graphics processing unit. Journal of biomedical optics.

[19] Huang Y-C, Ringold TL, Nelson JS, Choi B (2008). Noninvasive blood flow imaging for real-time feedback during laser therapy of port wine stain birthmarks. Lasers in Surgery and Medicine: The Official Journal of the American Society for Laser Medicine and Surgery.

[20] Nadort A, Kalkman K, Van Leeuwen TG, Faber DJ (2016). Quantitative blood flow velocity imaging using laser speckle flowmetry. Scientific reports.

[21] Schätzel K (1987). Correlation techniques in dynamic light scattering. Applied Physics B.

[22] Gisler T, Weitz DA (1999). Scaling of the microrheology of semidilute f-actin solutions. Physical review letters.

[23] Mason T, Ganesan K, Van Zanten J, Wirtz D, Kuo SC (1997). Particle tracking microrheology of complex fluids. Physical review letters.

[24] Sarmiento-Gomez E, Santamara-Holek I, Castillo R (2014). Mean-square displacement of particles in slightly interconnected polymer networks. The Journal of Physical Chemistry B.

[25] Hemar Y, Pinder D (2006). Dws microrheology of a linear polysaccharide. Biomacromolecules.

[26] Ochab-Marcinek A, Hołyst R (2011). Scale-dependent diffusion of spheres in solutions of flexible and rigid polymers: mean square displacement and autocorrelation function for fcs and dls measurements. Soft Matter.

[27] Erpelding M, Dollet B, Faisant A, Crassous J, Amon A (2013). Diffusing-wave spectroscopy contribution to strain analysis. Strain.

[28] Nagazi M-Y (2017). Space-resolved diffusing wave spectroscopy measurements of the macroscopic deformation and the microscopic dynamics in tensile strain tests. Optics and Lasers in Engineering.

[29] Robinson EA (1982). A historical perspective of spectrum estimation. Proceedings of the IEEE.

[30] Wiener N (1930). Generalized harmonic analysis. Acta mathematica.

[31] Lu, W. & Vaswani, N. The wiener-khinchin theorem for non-wide sense stationary random processes. *arXiv preprint arXiv:0904*.*0602* (2009).

[32] Bergland G (1969). A guided tour of the fast fourier transform. IEEE spectrum.

[33] Pieczywek PM, Cybulska J, Zdunek A, Kurenda A (2017). Exponentially smoothed fujii index for online imaging of biospeckle spatial activity. Computers and Electronics in Agriculture.

[34] Wang, D., Ranger, J. & Moyer, A. The spectral analysis of dynamic laser speckle patterns generated by brownian particle suspensions: A stroboscopic effect based filtering technique. *Advances in Optics* **2014** (2014).

[35] Zakharov, P., Cardinaux, F. & Scheffold, F. Accuracy preserving methods for faster measurements with dynamic light scattering and diffusing wave spectroscopy. In *Saratov Fall Meeting 2005: Coherent Optics of Ordered and Random Media VI*, vol. 6164, 61640K (International Society for Optics and Photonics, 2006).

[36] Zakharov, P. & Scheffold, F. Advances in dynamic light scattering techniques. In *Light Scattering Reviews 4*, 433–467 (Springer, 2009).

[37] Djaoui L, Crassous J (2005). Probing creep motion in granular materials with light scattering. Granular Matter.

[38] Takesue S, Mitsudo T, Hayakawa H (2003). Power-law behavior in the power spectrum induced by brownian motion of a domain wall. Physical Review E.

[39] Mo J, Simha A, Kheifets S, Raizen MG (2015). Testing the maxwell-boltzmann distribution using brownian particles. Optics express.

[40] Zakharov P, Völker A, Buck A, Weber B, Scheffold F (2006). Quantitative modeling of laser speckle imaging. Optics letters.

[41] Zakharov P (2009). Dynamic laser speckle imaging of cerebral blood flow. Optics express.

[42] Magatti D, Ferri F (2001). Fast multi-tau real-time software correlator for dynamic light scattering. Applied optics.

[43] Farraro R, Fathi O, Choi B (2016). Handheld, point-of-care laser speckle imaging. Journal of biomedical optics.

[44] Stewart C (2006). Kinetics of blood flow during healing of excisional full-thickness skin wounds in pigs as monitored by laser speckle perfusion imaging. Skin Research and Technology.

[45] Ruaro B (2014). Laser speckle contrast analysis: a new method to evaluate peripheral blood perfusion in systemic sclerosis patients. Annals of the rheumatic diseases.

[46] Strong AJ (2006). Evaluation of laser speckle flowmetry for imaging cortical perfusion in experimental stroke studies: quantitation of perfusion and detection of peri-infarct depolarisations. Journal of Cerebral Blood Flow & Metabolism.

[47] Stewart C (2005). A comparison of two laser-based methods for determination of burn scar perfusion: laser doppler versus laser speckle imaging. Burns.

[48] Forrester KR, Tulip J, Leonard C, Stewart C, Bray RC (2004). A laser speckle imaging technique for measuring tissue perfusion. IEEE transactions on biomedical engineering.

[49] Dunn AK, Bolay H, Moskowitz MA, Boas DA (2001). Dynamic imaging of cerebral blood flow using laser speckle. Journal of Cerebral Blood Flow & Metabolism.

[50] Lertsakdadet B (2018). Correcting for motion artifact in handheld laser speckle images. Journal of biomedical optics.

[51] Saggiomo V, Velders AH (2015). Simple 3d printed scaffold-removal method for the fabrication of intricate microfluidic devices. Advanced Science.

